# A randomized trial of artemether-lumefantrine and dihydroartemisinin-piperaquine in the treatment of uncomplicated malaria among children in western Kenya

**DOI:** 10.1186/1475-2875-12-254

**Published:** 2013-07-19

**Authors:** Aarti Agarwal, Meredith McMorrow, Peter Onyango, Kephas Otieno, Christopher Odero, John Williamson, Simon Kariuki, Stephen Patrick Kachur, Laurence Slutsker, Meghna Desai

**Affiliations:** 1 Malaria Branch, Division of Parasitic Diseases and Malaria, Centers for Disease Control and Prevention, Atlanta, GA, USA; 2 Kenya Medical Research Institute/Centers for Disease Control, Prevention Research and Public Health Collaboration, Kisumu, Kenya; 3 US Public Health Service, Rockville, Maryland

## Abstract

**Background:**

Artemether-lumefantrine (AL) was adopted as first-line treatment for uncomplicated malaria in Kenya in 2006. Monitoring drug efficacy at regular intervals is essential to prevent unnecessary morbidity and mortality. The efficacy of AL and dihydroartemisinin-piperaquine (DP) were evaluated for the treatment of uncomplicated malaria in children aged six to 59 months in western Kenya.

**Methods:**

From October 2010 to August 2011, children with fever or history of fever with uncomplicated *Plasmodium falciparum* mono-infection were enrolled in an *in vivo* efficacy trial in accordance with World Health Organization (WHO) guidelines. The children were randomized to treatment with a three-day course of AL or DP and efficacy outcomes were measured at 28 and 42 days after treatment initiation.

**Results:**

A total of 137 children were enrolled in each treatment arm. There were no early treatment failures and all children except one had cleared parasites by day 3. Polymerase chain reaction (PCR)-uncorrected adequate clinical and parasitological response rate (ACPR) was 61% in the AL arm and 83% in the DP arm at day 28 (p = 0.001). PCR-corrected ACPR at day 28 was 97% in the AL group and 99% in the DP group, and it was 96% in both arms at day 42.

**Conclusions:**

AL and DP remain efficacious for the treatment of uncomplicated malaria among children in western Kenya. The longer half-life of piperaquine relative to lumefantrine may provide a prophylactic effect, accounting for the lower rate of re-infection in the first 28 days after treatment in the DP arm.

## Background

Resistance to anti-malarials by *Plasmodium falciparum* has been an ongoing global public health concern since chloroquine resistance emerged in the 1960s [1]. Drug pressure is considered a major factor driving parasite resistance, as a result of the increased availability of medications in the public and private sectors, increased prevalence of sub-therapeutic drug concentrations, and counterfeit medications containing inadequate amounts of active ingredients, among other factors.

In Kenya, chloroquine was first-line treatment for uncomplicated *P. falciparum* malaria until 1998, despite the presence of high levels of chloroquine resistance since the early 1990s [2]. In 1998, sulphadoxine-pyrimethamine (SP) was adopted as first-line treatment, but resistance rapidly emerged. Both sulphadoxine and chloroquine have long half-lives and consequent prolonged parasite exposure to subtherapeutic drug levels, which can contribute to resistance.

Artemisinins, the newest class of anti-malarials, have a very short half-life (<8 hours) and rapidly reduce parasite burden; their use in combination with other anti-malarials decreases the chance of resistance emergence [3]. However, artemisinin resistance has developed along the Thai-Cambodian border, likely due to its use at sub-therapeutic doses and as monotherapy [4]. Artemisinin-based combination therapy (ACT) has superior efficacy and the potential to prevent drug resistance by incorporating a partner drug to enhance parasite clearance. ACT has been adopted as first-line treatment in most malaria-endemic countries [5]. Artemether-lumefantrine (AL), a highly effective ACT, is commonly used in many African countries as first-line treatment [6]. In 2004, Kenya adopted AL as first-line malaria treatment, but it was not widely implemented until 2006 [7].

Dihydroartemisinin-piperaquine (DP), another ACT regimen, has been studied in East Africa as an alternative to AL [8-10]. Its advantages over AL include once-daily dosing and a longer half-life of the partner drug, which may prevent re-infection in areas of intense malaria transmission. Studies have shown equivalent safety and efficacy profiles for DP and AL [10-12]. In 2010, DP was adopted as second-line treatment for uncomplicated *P. falciparum* malaria in Kenya.

Regular monitoring of anti-malarial efficacy is essential to better inform national malaria policies [13]. This study was conducted in western Kenya to determine if AL remains efficacious for the treatment of uncomplicated malaria after five years of its implementation and to evaluate the efficacy of DP in this population.

## Methods

### Study site and enrolment

This study was conducted between October 2010 and August 2011 at Siaya District Hospital (SDH) in Nyanza Province, western Kenya. This region is holoendemic for *P. falciparum* with high malaria transmission and two seasonal peaks, April to July and November to December. The entomological inoculation rate (EIR) in this area, historically around 300 infectious bites per person per year, has recently been estimated to be ten infectious bites per person per year (Gimnig J, pers comm). Study subjects were recruited from the outpatient paediatric department of SDH, which serves approximately 100 patients per day.

### Subjects

Children aged six to 59 months with *P. falciparum* mono-infection were enrolled. Additional inclusion criteria were axillary temperature ≥37.5°C or history of fever in the previous 48 hours, weight ≥5.0 kg, parasitaemia 1-200,000 asexual forms per μL (initially 2,000-200,000 but protocol amended in January 2011 to include any parasitaemia <200,000 as these drug regimens are used to treat patients with any level of parasitaemia in Kenya), residence within 10 km of SDH, and written informed consent by caregiver. Subjects were excluded if any of the following were present: lethargy, convulsions, inability to drink, persistent vomiting, symptoms of severe malaria, severe malnutrition (weight-for-age ≤3 standard deviations below the mean for gender according to World Health Organization (WHO) standards), severe anaemia (haemoglobin (Hb) <5 g/dl), known hypersensitivity to study drugs, presence of febrile illness other than malaria (e.g. measles, pneumonia), presence of chronic medical conditions, treatment with any anti-malarial in the previous two weeks, or previous enrolment in any malaria study.

### Ethical considerations

This study received ethical clearance from the US Centers for Disease Control and Prevention (CDC, Atlanta, USA) and the Kenya Medical Research Institute (KEMRI, Nairobi, Kenya). Written informed consent was obtained from caregivers of enrolled subjects and a long-lasting insecticide-treated bed net (ITN) was provided to enrolled subjects.

### Clinical and laboratory procedures

This was a 42-day, open-label *in vivo* trial [14]. Initial screening was offered to patients with fever or history of fever. Caregivers were then asked about interest in study participation. After consenting patients were screened for inclusion criteria, a rapid diagnostic test (RDT) (SD Bioline malaria Pf/pan, Standard Diagnostics Inc, Yongin, South Korea) for malaria and Hb testing (Hemocue® Hb 201+, Hemocue AB, Angelholm, Sweden) were performed. If the RDT was positive and Hb was ≥5.0 g/dL, two thick and thin blood films were collected to assess parasitaemia and confirm malaria species. Blood films were read independently by two microscopists by counting the number of asexual parasites against 500 white blood cells (WBCs). Slides were considered to be negative only after examining fields containing 1,000 WBCs. The geometric mean of the two readings was considered in the analyses. Slides with parasite densities discordant by more than 50% or with positive and negative results were re-examined by a third microscopist; the mean of the third read and the closest of the first two slides was considered final. All microscopists were blinded to the treatment arm and were certified as expert readers through a quality assurance programme at the South African National Institute for Communicable Diseases.

Caregivers of enrolled children were interviewed and children were examined by a study clinician. Children were block randomized in fixed blocks of ten to treatment with AL (Coartem®; Novartis, Basel, Switzerland) or DP (DuoCotexin®; Holley-Cotec Pharmaceuticals, Beijing, China). Samples of the AL and DP used in this study were sent to CDC laboratories for quality testing using high-performance liquid chromatography (HPLC) (Agilent Technologies, Waldbronn, Germany). Both treatments were co-formulated, fixed-dose ACT regimens and were administered under direct observation by study staff at the study clinic (except AL evening doses) for three consecutive days. AL tablets, consisting of 20 mg of artemether and 120 mg lumefantrine, were administered twice daily according to patient weight: 5-14 kg: one tablet per dose; weight 15-24 kg: two tablets per dose; weight 25-34 kg: three tablets per dose. Morning doses were given with milk and directly observed in the study clinic. Caregivers were given evening doses to administer at home with food or milk. DP tablets, consisting of 20 mg dihydroartemisinin and 160 mg of piperaquine phosphate, were administered once daily by study staff according to patient weight: 5-6 kg: one-half tablet daily; 7-9 kg: one tablet daily; 10-14 kg: two tablets on day 0, then one tablet on days 1 and 2; 5-19 kg: two tablets daily. A full dose was re-administered if the patient vomited within 30 min or a half dose if vomiting occurred between 31 and 60 min. Patients with vomiting within 30 min of the second dose were referred for parenteral treatment and withdrawn from the study.

### Follow up

Children were followed for 42 days and asked to return on days 1, 2, 3, 7, 14, 21, 28, 35, and 42 following enrolment, as well as any day if ill. The study clinic was open daily during regular hours; study personnel provided after-hours care at SDH. A clinical assessment was performed and blood smears were collected at each study visit. Hb levels were measured on days 0, 7, 14, 28, and 42. A filter paper blood spot was collected on days 0, 3, and 7 and in case of suspected treatment failure for molecular analysis. Adverse events were investigated and addressed.

### Outcomes

Efficacy was assessed by clinical and parasitological outcomes using WHO definitions [14]. Children were classified as early treatment failure (ETF) if any of the following criteria were met: development of severe malaria by day 3, day 2 parasitaemia > day 0 parasitaemia, presence of parasites on day 3 with axillary temperature ≥37.5°C, or day 3 parasitaemia >25% of day 0 parasitaemia. Children not meeting ETF criteria with *P. falciparum* parasitaemia occurring between day 7 and 28 or 42 without fever were classified as late parasitological failure (LPF). Those with fever occurring between day 4 and day 28 or day 42 with parasitaemia were classified as late clinical failure (LCF). If no failure was recorded by day 28 or day 42, the outcome was classified as adequate clinical and parasitological response (ACPR). All treatment failures with uncomplicated malaria were treated with AL and treatment failures with severe malaria were treated with parenteral quinine. Follow-up ended once a study subject met one of the four classification criteria: ETF, LPF, LCP or ACPR.

### Molecular analysis

To differentiate between recrudescence and re-infection, a genotypic analysis based on merozoite surface protein-2 (*msp2*), glutamate-rich protein (*glurp*), and merozoite surface protein-1 (*msp1*) was performed by PCR using filter-paper blood spots [15]. Recrudescence was defined as at least one identical allele for each of the three markers (*msp2, glurp*, and *msp1*) in the pre- and post-treatment samples.

### Statistical analysis

Primary efficacy outcomes included day 28 and day 42 ACPR, both PCR-corrected and PCR-uncorrected for each ACT regimen. Secondary outcomes included haematologic response, rates of fever clearance and parasite clearance by day 3, rates of ETF, LPF and LCF. Assuming a PCR-corrected ACPR of 95% and 20% loss to follow up at 42 days, a sample size of 137 children per study arm was chosen, 274 children in total; this allowed for a precision rate of +/- 4.5% at a 5% significance level. This study was not powered to detect a difference in efficacy between treatment arms.

Study forms were scanned into a Microsoft Access 2000 (Microsoft, Redmond, USA) database. Statistical analysis was performed using SAS® 9.2 (SAS Institute, Cary, USA). Per protocol (PP) analysis of outcomes excluded those children withdrawn from the study for any reason. Intention-to-treat (ITT) analysis was performed using survival analysis. Kaplan-Meier curves were estimated for both 28 and 42 days of follow up; the log-rank test was used for comparing the curves. For ITT analysis, all withdrawals, losses to follow up and treatment failures were censored on the last day of follow up. Comparisons were made using χ^2^ test for categorical variables and Student’s t-test or Wilcoxon rank-sum test (for non-parametric data) for continuous variables. A two-sided p-value <0.05 was considered statistically significant.

## Results

### Baseline characteristics

**Figure 1:**
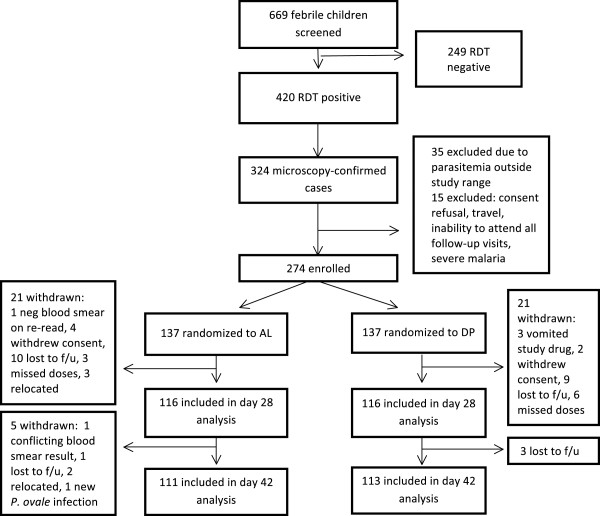
**Trial profile, western Kenya, 2011.** Legend: RDT: Rapid diagnostic test for malaria; f/u: follow up; neg: negative.

A total of 669 children with fever or history of fever were screened (Figure 1). Among those, 420 (63%) were RDT-positive and 324 (77%) of RDT-positive children had microscopy-confirmed *P. falciparum* mono-infection. Of these remaining 324 eligible children, 50 (15%) did not meet other inclusion criteria or did not give consent. Two hundred and seventy-four children were enrolled in the study, 137 in each arm. Among those enrolled, 224 were included in the day 42 analysis, 111 in the AL arm and 113 in the DP arm. Baseline characteristics of the children enrolled in the two arms were similar (Table 1). The percentage of children withdrawn from analysis was similar between the two arms, 18.2% in AL arm and 17.5% in the DP arm (p = 0.88). Reasons for withdrawal are shown in Figure 1.

**Table 1:** Baseline characteristics of children upon enrolment for artemether-lumefantrine (AL) and dihydroartemisinin-piperaquine (DP), western Kenya 2011

**Characteristic**	**AL (n = 137)*(95% CI)***	**DP (n = 137)*(95% CI)***	**p-value**
**Mean age** (months)	36.1 (33.8–38.5)	33.5 (31.1–35.9)	0.11
**Male** (%)	55	57	0.9
**Mean weight** (kg)	13.3 (12.8–13.8)	13.0 (12.5–13.4)	0.3
**Mean axillary temperature** (°C)	37.6 (37.4–37.8)	37.7 (37.5–38.0)	0.35
**Mean haemoglobin** (g/dL)	9.7 (9.4–10.0)	9.9 (9.7–10.2)	0.28
**Geometric mean day 0 parasite density** (parasites/ μL) (range)	45,168 (34,506–47,190) (10–148,027)	49,248 (35,188–52,544) (54–166,584)	0.49

### Clinical and parasitological outcomes

No ETF was observed in either treatment arm (Table 2). Follow up was completed for 116 children in each arm up to day 28. PCR-uncorrected ACPR was 61% in the AL arm and 83% in the DP arm (p = 0.001). PCR-uncorrected results showed day 28 LCF and LPF were 11% and 28% in the AL arm, respectively; in the DP arm, day 28 LCF and LPF were 3% and 14%, respectively. PCR analysis revealed the majority of LCF and LPF cases in both arms were due to re-infection, 34 (92%) of 37 in the AL arm and 18 (95%) of 19 in the DP arm. However, the re-infection rate by day 28 in the DP arm was significantly lower than that of the AL arm (p = 0.03). At day 28, PCR-corrected ACPR was 97% for AL and 99% for DP (p = 0.5). The nine children for whom PCR results were missing due to lost filter-paper samples were excluded from the PCR-corrected analysis (eight from the AL arm and one from the DP arm).

**Table 2:** Clinical and parasitological response rates for artemether-lumefantrine (AL) and dihydroartemisinin-piperaquine (DP) using per protocol analysis, western Kenya 2011

**Outcome**	**AL**	**DP**	**p-value**
	**% (95% CI)**	**% (95% CI)**	
**Early treatment failure**	0% (0%–3%) (0/137)	0% (0%–3%) (0/137)	1
**Day 3 parasite clearance**	99% (96%–99%) (130/131)	100% (97%–100%) (126/126)	0.34
Day 28 PCR-uncorrected ACPR^*^	61% (52%–70%) (71/116)	83% (75%–89%) (96/116)	0.001
Day 28 PCR-corrected ACPR^**^	97% (92%–99%) (105/108)	99% (95%–100%) (114/115)	0.48
**Day 42 PCR-uncorrected ACPR**	44% (35%–54%) (49/111)	54% (45%–63%) (61/113)	0.14
**Day 42 PCR-corrected ACPR**	96% (90%–99%) (97/101)	96% (91%–99%) (105/109)	0.26

*Polymerase chain reaction-uncorrected adequate clinical and parasitological response.

**Polymerase chain reaction-corrected adequate clinical and parasitological response.

For day 42, analysis was completed for 111 children in the AL arm and 113 children in the DP arm. The PCR-uncorrected ACPR was 44% for the AL arm and 54% for the DP arm (p = 0.14). PCR-uncorrected results showed that the day 42 LCF and LPF were 18% and 37% in the AL arm, respectively; while LCF was 10% and LPF was 36% in the DP arm. The re-infection rate was similar for the two arms at day 42 (p = 0.7). After PCR correction, ACPR for both arms was 96%. Similarly, the children for whom PCR results were missing were excluded from analysis (ten in AL arm and four in DP arm).

To ensure the results of the PCR-corrected analysis were not biased by missing samples, sensitivity analyses were performed assuming all missing PCR results were due to re-infection and all were due to recrudescence, resulting in a range of PCR-corrected ACPR. For day 28, the maximum ranges of PCR-corrected ACPR for the treatment arms are 91-97% (95% CI: 85-100%) in the AL arm and 98-99% (95% CI: 96-100%) in the DP arm. For day 42, ACPR would be 87-96% (95% CI: 81-100%) in the AL arm and 92-96% (95% CI: 87-100%) in the DP arm.

**Figure 2:**
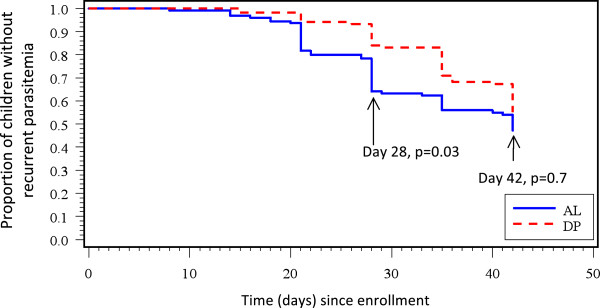
**Survival curve of enrolled children by PCR-uncorrected data, western Kenya 2011.** Legend: This graph is using intention to treat (ITT) analysis.

At day 28, the survival analysis using the ITT definition demonstrated a PCR-uncorrected cure rate of 67% for AL and 85% for DP (p = 0.0004) (Figure 2). At day 42, the PCR-uncorrected cure rates were 55% for AL and 62% for DP (p = 0.22). The PCR-corrected cure rates at day 28 were 98% for AL and 99% for DP (p = 0.28). At day 42, PCR-corrected cure rates were 97% for both drugs.

### Laboratory outcomes

Treatment with either AL or DP resulted in rapid parasite clearance. Although >75% of children in both arms remained parasitaemic on day 1, only four (3%) and five (4%) children remained parasitaemic on day 2 in the AL and DP arms, respectively. One child in the study remained parasitaemic on day 3 (AL arm). Over 90% of children were afebrile by day 1 in both treatment arms. Mean Hb of children who were not re-infected increased from a baseline of 9.8 g/dL to 11.6 g/dL at day 42, whereas the mean Hb of those re-infected increased from a baseline of 9.9 g/dL to 11.1 g/dL on the last study day (p = 0.9). The change in Hb from baseline to the study endpoint was similar among the two study arms. Drug samples tested for quality assurance contained adequate concentrations of active ingredients.

### Adverse events

There were three children in the DP arm who vomited the drug twice following enrolment and were referred for alternative treatment. The rates of vomiting for the first dose of medication were similar for AL and DP (3.7% and 4.9%, respectively) and not associated with age.

Three enrolled children developed severe malaria more than 28 days after treatment; two in the AL arm and one in the DP arm. These children were hospitalized for parenteral treatment. These outcomes are attributable to re-infection (confirmed by PCR analysis), not poor efficacy of treatment regimens. All children recovered completely; no deaths occurred during the study.

## Discussion

Both AL and DP are efficacious in treating uncomplicated *P. falciparum* malaria in children in western Kenya. Recurrent parasitaemia among children under five years is frequent in this area despite high coverage with ITNs (70% household ownership and 42% usage in children under five years) [16] and is mostly secondary to re-infection with *P. falciparum*. As seen in other African countries, recurrent parasitaemia occurs significantly more frequently in those children treated with AL in the first 28 days [8,9,11]. This is likely due to the longer half-life of the piperaquine component of DP, which provides long-lasting prophylactic effect. This study was not powered to compare the efficacy of the two drug regimens; however, there was a significant difference in re-infection at day 28 between the two groups.

Data from similar studies conducted in western Kenya from 2005 to 2009 assessing the efficacy of AL and DP had similar results. Data collected in 2005 on children with uncomplicated malaria and treated with AL showed PCR-uncorrected ACPR at day 28 of 71% and at day 42 of 41%, compared to 61% and 44% in this study [17]. However, the high numbers of recurrent parasitaemia were likely due to re-infection and not recrudescence, as it was the case in this and other studies [18]. A smaller study conducted in 2007, showed that only one (1.5%) out of 67 patients had recurrent parasitaemia at day 28 after treatment with AL and no episodes of recurrent parasitaemia were detected at day 28 in the DP group [19]. When comparing these data to the higher rates of recurrent parasitaemia in our study, we do not believe it is due to declining drug efficacy, but rather to the high frequency of re-infection with *P. falciparum* in children in the study area. Lastly, data collected in 2009 in the Mbita region of western Kenya had similar PCR-corrected ACPR in the AL group (98.6% at day 28 and 97.2% at day 42) as what was observed in this evaluation, which further demonstrates the continued efficacy of AL in the region [18].

There is growing concern about the emergence of artemisinin resistance, signalled by delayed parasite clearance, as observed in Southeast Asia [20]. One recent study from the Kenyan coast reports decreased parasite clearance rates in those treated with ACT since 2006 [21]. This study showed that although over 75% of children remained parasitaemic on day 1, over 95% had cleared parasitaemia by day 2, and only one child had parasites on day 3. These clearance rates are similar to those seen in an efficacy study with AL conducted in this region in 2006 [22]. As WHO defines suspected artemisinin resistance as ≥10% of cases with parasitaemia at day 3 after ACT treatment initiation [23], this study provides no evidence to suggest artemisinin resistance in western Kenya.

Although the annual EIR in western Kenya has decreased dramatically, from 300 infectious bites per person per year in 1990 [24] to ten in 2010, malaria prevalence during the peak transmission season in children under five years of age remains high at 42% (KEMRI/CDC, unpublished data) [25]. A previous study demonstrated that in several areas in Africa with annual EIRs in the range of one to ten infectious bites per person per year, the mean parasite prevalence remained similarly high [26]. In fact, parasite prevalence was not meaningfully reduced until the annual EIR was well below one infectious bite per person per year. The high parasite prevalence despite low EIR is multifactorial. Some plausible reasons include recurrent infections due to *Plasmodium vivax* and *Plasmodium ovale*, lack of access to care resulting in delayed treatment, asymptomatic carriers, and frequent anti-malarial stock-outs [27]. This epidemiological context likely accounts for the high re-infection rates observed in this study, despite a large reduction in EIR over the past two decades.

Both drug regimens were well tolerated with a low frequency of vomiting. Although the frequency of vomiting the first dose was similar between the two treatment arms, only those in the DP arm (n = 3) vomited the drug twice and required alternative treatment. Increased vomiting in children aged six to 24 months with uncomplicated malaria treated with DP, compared to AL, has been noted previously [28]. Vomiting did not seem to be related to younger age in either group. However, this study was not powered to detect such a difference.

There are a few limitations to this study. First, the evening doses of AL were not directly observed. Although caregivers were asked to confirm administration of evening doses and children who missed doses were withdrawn, the efficacy of AL may be underestimated in this study if some missed doses were not reported. Similar studies have used the same approach [29]. However, PCR-corrected efficacy of AL was still high in this study. In addition, PCR data are missing for some children with treatment failure. Nevertheless, as recrudescence was responsible for <10% of failures in both arms, the ACPRs calculated considering only available PCR samples are likely accurate. Also, parasitaemia range for study inclusion was altered to include all children with *P. falciparum* mono-infection. This increased the risk of including false positives; however, only certified microscopists read slides and only nine subjects had parasitaemia <2,000 parasites/μL. Lastly, contrary to the original protocol, two months after initiation of recruitment, study staff excluded some children with any history of vomiting. The number of children excluded for this reason is unknown, as only persistent vomiting was an original exclusion criterion. Therefore, this study may underestimate the incidence of vomiting associated with the treatment regimens, as well as inadvertently excluding some children with high parasitaemia or high fever.

DP may benefit children in this high-transmission setting because the long half-life of piperaquine is associated with a decreased re-infection rate in the first 28 days after treatment compared to AL. This longer prophylactic effect may allow more time for Hb recovery, thus decreasing the severity of re-infections. The once-a-day dosing is another advantage of this regimen and may improve adherence. However, the higher cost of a treatment dose of DP compared to AL, US$4 and US$1, respectively, may be a barrier to its use as first line. In addition, DP may be more prone to the development of resistance because of the long half-life of piperaquine.

## Conclusions

The results of this study demonstrate that AL and DP remain efficacious treatment regimens for uncomplicated *P. falciparum* malaria in western Kenya. With day 3 parasite clearance rates of nearly 100%, there is no evidence of delayed parasite clearance to indicate emerging artemisinin resistance. Following WHO recommendations, regular monitoring to evaluate anti-malarial efficacy at least every two years should be maintained to confirm the continued efficacy of first-line anti-malarial therapy.

## Competing interests

The authors declare that they have no competing interests.

## Authors’ contributions

All authors contributed to the design of the study and assisted with data interpretation. KO carried out the molecular genetic studies. MM, MD, LS, SK and SPK conceived of the study, and participated in its design and coordination as well as manuscript production. AA participated in the design and coordination of the study, performed data analysis and drafted the manuscript. PO and CO participated in the coordination of the study and data analysis. JW contributed to the design of the study and assisted in statistical analysis. All authors read and approved the final manuscript.
